# Intravital Assessment of Cells Responses to Conducting Polymer-Coated Carbon Microfibres for Bridging Spinal Cord Injury

**DOI:** 10.3390/cells10010073

**Published:** 2021-01-05

**Authors:** Bilal El Waly, Vincent Escarrat, Jimena Perez-Sanchez, Jaspreet Kaur, Florence Pelletier, Jorge Eduardo Collazos-Castro, Franck Debarbieux

**Affiliations:** 1 Institut des Neurosciences de la Timone (UMR7289), Aix-Marseille Université and Centre National de la Recherche Scientifique, 13005 Marseille, France; bilal.el-waly@univ-amu.fr (B.E.W.); vincent.ESCARRAT@univ-amu.fr (V.E.); jimenara@gmail.com (J.P.-S.); Jaspreetkaur1306@gmail.com (J.K.); florence.pelletier@univ-amu.fr (F.P.); 2 Centre Européen de Recherche en Imagerie Médicale, Aix-Marseille Université, 13005 Marseille, France; 3 Department of Neuroscience, University of Copenhagen, 2200 Copenhagen N, Denmark; 4 Neural Repair and Biomaterials Laboratory, Hospital Nacional de Parapléjicos (SESCAM), 45071 Toledo, Spain; 5 Institut Universitaire de France, 75005 Paris, France

**Keywords:** dorsal hemisection, transgenic multifluorescent mice, microfibre scaffold, axonal regeneration, two photon imaging, microglia

## Abstract

The extension of the lesion following spinal cord injury (SCI) poses a major challenge for regenerating axons, which must grow across several centimetres of damaged tissue in the absence of ordered guidance cues. Biofunctionalized electroconducting microfibres (MFs) that provide biochemical signals, as well as electrical and mechanical cues, offer a promising therapeutic approach to help axons overcome this blind journey. We used poly(3,4-ethylenedioxythiophene)-coated carbon MFs functionalized with cell adhesion molecules and growth factors to bridge the spinal cord after a partial unilateral dorsal quadrant lesion (PUDQL) in mice and followed cellular responses by intravital two-photon (2P) imaging through a spinal glass window. Thy1-CFP//LysM-EGFP//CD11c-EYFP triple transgenic reporter animals allowed real time simultaneous monitoring of axons, myeloid cells and microglial cells in the vicinity of the implanted MFs. MF biocompatibility was confirmed by the absence of inflammatory storm after implantation. We found that the sprouting of sensory axons was significantly accelerated by the implantation of functionalized MFs after PUDQL. Their implantation produced better axon alignment compared to random and misrouted axon regeneration that occurred in the absence of MF, with a most striking effect occurring two months after injury. Importantly, we observed differences in the intensity and composition of the innate immune response in comparison to PUDQL-only animals. A significant decrease of immune cell density was found in MF-implanted mice one month after lesion along with a higher ratio of monocyte-derived dendritic cells whose differentiation was accelerated. Therefore, functionalized carbon MFs promote the beneficial immune responses required for neural tissue repair, providing an encouraging strategy for SCI management.

## 1. Introduction

Significant progress has been made in the understanding of fundamental processes that occur after spinal cord injury (SCI), including the involvement of multiple cell types, inflammatory reactions and the expression of trophic factors [1,2]. The irreversible functional impairment after SCI is caused by the disruption of neuronal connections across the lesion and the generation of refractory substrates that inhibit spontaneous recovery [3,4]. However, the discovery of the regenerative capacity of central nervous system (CNS) neurons in the proper environment, and the beneficial effects of their electrical stimulation, gives reasonable optimism that solutions for repair could soon be found. Indeed, despite the limited neuronal regeneration after SCI, some sprouting of axon collaterals does ensue [5,6,7,8]. Axons undergo remarkable structural rearrangements, and this property may underlie the recovery of lost functions that occur in some incomplete injuries [4,9,10]. Even then, axonal sprouting does not replicate the exact connectivity of axons prior to injury, and targeting defects occur. These ectopic axonal projections produce improper rewiring, which may give rise to maladaptive plasticity and severe functional deficits [8]. Furthermore, the lesion that is formed, and its refractory environment, lack the necessary substrate for axon regrowth and guidance. A major aim of SCI research is to repair the disrupted neural network to close to its former status. Moreover, the regenerative capabilities of electrical stimulation on CNS tissue have been well studied [11] and the acceleration of axon outgrowth has been reported [5,12]. Therefore, it is becoming increasingly more frequent to use combined strategies that enhance regrowth and guidance.

Biomaterial scaffolds that provide guidance and a growth-permissive substrate for axons are logical therapeutic options. Many different types of scaffolds have been developed for the treatment of SCI, which provide biophysical and/or biochemical cues that support the regenerative process [13,14]. The ideal scaffold would have a simple design that allows for smooth manufacturing, low immunogenicity, mechanical properties that prevent mismatching with the neural tissue, biochemical cues to favor cell adhesion and axonal regeneration, and would be easy to transplant into the injured spinal cord not to entail further damage [13,15]. In particular, scaffolds made of conducting polymers, such as poly 3,4-ethylenedioxythiophene (PEDOT) have been shown to be suitable candidates for the fabrication of devices for neural repair [16]. Indeed, PEDOT doped upon carbon microfibres (MFs) increased their electrical conductivity and provided a substrate to attach bioactive molecules that promote longitudinal alignment of glial cells and axonal elongation [17,18]. Since the microenvironment of the SCI lesion inhibits regeneration, we hypothesised that when coated, these MFs may also provide a chemical environment that favours growth.

In the present study, we implemented a partial unilateral dorsal quadrant lesion (PUDQL) model of SCI in mice with a glass window-based *in vivo* imaging methodology. The lesion is compatible with the implantation of carbon MFs, functionalized with PLL/heparin/bFGF/fibronectin. We used Thy1-CFP//LysM-EGFP//CD11c-EYFP triple transgenic reporter animals [19,20] that allowed simultaneous imaging of axons, myeloid cells and microglial cells over time in the same animal to monitor the effects of the grafted MFs on the dynamics of axon regrowth and inflammation. We found that functionalized MFs accelerated and optimized the recovery of axonal network after PUDQL, likely by dampening chronic inflammation and promoting the accumulation of monocytes derived dendritic cells (moDCs).

## 2. Materials and Methods

### 2.1. Animals

We operated on a total of n = 43 Thy1-CFP//LysM-EGFP//CD11c-EYFP triple heterozygous transgenic adult mice (more than nine weeks old) with multiple fluorescent cell populations. These mice display a subpopulation of neurons with cytoplasmic expression of CFP (blue), neutrophils and monocytes with cytoplasmic expression of Enhanced Green Fluorescent Protein (EGFP), as well as activated resident microglia and peripheral dendritic cells with cytoplasmic expression of Enhanced Yellow Fluorescent Protein EYFP (see 20 for details). Mice were housed in cages with food and water ad libitum in a 12 h light/dark cycle at 22 ± 1 °C. Until the end of the protocols, food was supplemented with 4% agarose jelly containing 4%glucose. All experimental procedures were performed in accordance with the French legislation and in compliance with the European Community Council Directive of 24 November 1986 (86/609/EEC) for the care and use of laboratory animals. The research on animals was authorised by the Direction Départementale des Services Vétérinaires des Bouches-du-Rhône (license D-13-055-21) and approved by the National Committee for Ethic in Animal Experimentation (Section N°14, project APAFIS #4828-20 1504131125423 v2).

### 2.2. Preparation and Biofunctionalization of Carbon MFs

The protocol for the preparation and bio-functionalization of carbon MFs was described previously [17,18,21]. Briefly, poly(3,4-ethylenedioxythiophene)/poly-(styrenesulfonate)-co-maleic acid) (PEDOT:PSS-co-MA) were electrodeposited on the surface of 7-µm-diameter carbon fibres (Goodfellow) by applying a constant anodic current of 1 µA/mm^2^ and a polymerization charge of 96 mC/cm^2^. Functionalisation with bioactive molecules was performed on PEDOT:PSS-co-MA-coated carbon MFs (hereafter referred to as bare-MFs). Poly-L-lysine (PLL; Sigma-Aldrich) was bonded covalently to the carboxylic groups of the dopant. Heparin (10 mM; Sigma-Aldrich) was dissolved in Phosphate Buffer Solution (PBS) was then assembled on the PLL layer for 4 min. Subsequently, recombinant human basic fibroblast growth factor (bFGF, PeproTech 100-18B) was applied at 1 µg/mL in PBS for 1 h. Finally, the MFs were incubated at 37 °C for 4 days with bovine fibronectin at 40 µg/mL (FN; Invitrogen, 33010-018) dissolved in PBS, as illustrated in Collazos Castro et al. [17]. PEDOT/PLL/Heparin/bFGF/FN-MFs are hereafter referred to as MFs, or eventually as bf-MFs (for biofunctionalized-bf-MFs) when these were compared to bare MFs in Section 3.2.

### 2.3. Partial Unilateral Dorsal Quadrant Lesion (PUDQL) and Glass Window Implantation

A glass window was cemented and sealed on the exposed spinal parenchyma [19]. Briefly, mice were anesthetized with intraperitoneal ketamine/xylazine (120 mg/kg; 12 mg/kg), and supplemented hourly with the same cocktail at a lower dose (40 mg/kg; 4 mg/kg). Following a dorsal midline incision over T12 to L2, the muscles between the spinal and transverse processes were resected using a scalpel. Animals were then suspended from a spinal-fork stereotaxic device (Harvard Apparatus). The dorsal musculature was further resected to expose the vertebrae and the tips of modified staples were inserted along the edges of the T12 and L2 vertebrae and glued in place with cyanoacrylate. A modified paperclip was fixed to the staples to serve as a handle for surgery and imaging, and a layer of dental cement was applied to form a rigid ring to hold the vertebrae, staples and paper clip together. A laminectomy was performed by removing two spinal processes from the exposed vertebrae.

A partial unilateral dorsal quadrant lesion (PUDQL) was made on the rostral edge of the exposed spinal cord. To do so, a 26G needle blade was used to make a 0.6 mm incision on the right side of the spinal cord as close as possible to the central vein and the spinal cord, as deep as the shaft of the needle. The spinal cord was then transected using a pair of fine-angled Vannas spring scissors (Fine Science Tools) from the rostral end of the incision to 0.4 mm lateral and approximately 0.5 mm deep from the dorsal surface. A second identical incision was made from the caudal end of the initial incision. A final horizontal cut was made from the injured side toward the dorsal vein and the tissue between the rostral and caudal transections was removed. The lesion was made so that the caudal incision reached the middle of the window and the rostral incision was 200 microns away from the most frontal edge of the window. Special care was taken to not cut the central vein so that bleeding was kept minimal. Animal were split into two groups: PUDQL only and PUDQL immediately followed by MF implantation.

In MF-implanted animals, four carbon bf-MFs aligned in parallel to the central vein were inserted into the spinal cord parenchyma to bridge the injury. To evaluate the influence of protein coating, a subset of animals were implanted with two bare-MFs and two bf-MFs in alternation. In this case, difference in biocompatibility and on axon sprouting were assessed.

A line of liquid Kwik-Sil (World Precision Instruments) was applied along the midline of the spinal cord, and the glass window was immediately glued and cemented over the spinal cord. Kwik-Sil was used to embed the MFs and seal the lesion site to the glass window in order to prevent fibroblastic invasion. Kwik-Sil was applied on the dried lesion site using the mixing application tip. The glass window had to be perfectly clean to allow adherence between the silicone and the glass. All the procedures were performed under sterile conditions. Postoperative analgesia was obtained by administration of cortamethasone (0.2 mg/kg) and rimadyl (5 mg/kg) immediately following surgery and every two days for 10 days after surgery. Mice were active in their cage already 18h postsurgery and did not require manual bladder emptying, since the lesion affected only the most dorsal tracts.

### 2.4. Intravital Imaging

The same mice were imaged three days (D3) after window implantation to target the peak of peripheral cell infiltration and then every two weeks for two to three months (D15, D28-30, D42-45, D58-60, D90). For each imaging session, mice were lightly anesthetized with 1.5% isofluorane (*v/v*) in air for 2 min, followed by intraperitoneal ketamine/xylazine (100 mg/kg; 10 mg/kg) administration. Animals were supplemented with 0.4–1.0% isofluorane (*v/v*) in air from 45 min after the start of the session until completion. Throughout imaging, animals were freely breathing and the microscope chamber was warmed to 32 °C to maintain the body temperature at 37 °C. Following each imaging session, the animals were returned to their cage with a piece of tissue for nesting and kept warm until they recovered from anesthesia.

A tunable femtosecond pulsed laser (Ultra II Chamaleon Coherent) was coupled to a Zeiss two-photon (2P) microscope (LSM 780) equipped with a 20× water immersion objective lens (NA = 1.0) and five non-descanned detectors. The laser was tuned to 940 nm to optimize the simultaneous excitation of the labeling fluorophore combination while minimizing the heat accumulation by implanted carbon MFs. Filter sets were designed to optimize the separation of the emission spectra of multiple fluorophores. For each image stack laser intensity was adjusted according to imaging depth in order to maximize signal intensity while minimizing saturation throughout the image stack.

A second harmonic signal reflected back by superficial collagen fibres was used to identify meninges. Blood vessels and remarkable axons patterns were used as anatomical markers to find the region of interest for each animal. Tiled-stack images were acquired with a field of view of 424 × 424 µm with an optical sectioning of 3 µm over a depth of ~80 to 100 µm below the meninges. Microadjustments during mouse positioning allowed extraction of a conserved volume of interest throughout imaging sessions. The volume mainly lying between 20 to 50 µm was used for quantitative analysis.

### 2.5. Image Analysis

Images were analyzed using ZEN 2.1 (Zeiss, Jena, Germany), ImageJ software and Arivis Vision 4D software (Arivis AG, Berlin, Germany). ZEN 2.1 was used for post-acqusition spectral unmixing and analysis was performed on the resulting data. Presented images are pseudocolored and contrast-enhanced for clarity. For every mouse, the volumes of interest imaged at every time point were manually registered to account for small inaccuracy in repositioning both in the XY plane and in the Z position from one session to the next. For every time point in this registered volume of interest, axons were counted manually in 3D at five locations along the rostro-caudal axis, at the level of the lesion epicenter and respectively 400 µm and 800 µm in the caudal direction as well as 400 µm in the rostral direction. Counting was done on both sides of the dorsal vein. The numbers obtained on the lesioned ipsilateral side were normalized by the corresponding numbers of axons on the uninjured contralateral side. To study the coating dependance of the density of axons found around MFs, we evaluated the number of axons intercepting three different 100 µm segments running perpendicularly to the MF surface at three different positions along the MF segment visible in the centre of the lesion. Axons enclosed in the area between bare MF and bf-MFs were likely submitted to a mixed influence of both MF and were therefore not included in the counting.

Immune cells were counted in the total 3D volume of the 2P acquisitions using the object detection algorithm in the Arivis software, and their average densities were evaluated per volume unit (of about 0.01 mm^3^). Cells were distinguished using EGFP and EYFP labeling and morphological features. To validate our automated procedure, automated results were compared to manual counts of cellular densities obtained by two independent investigators on selected images stacks; these never differed by more than a few percent.

### 2.6. Statistics

All data are expressed as mean ± SEM. Statistical analysis was performed using Graph Pad Prism software for Kruskal-Wallis or Mann-Whitney tests, as required. *p* < 0.05 was considered for statistical significance.

### 2.7. Nissl Staining

Mice were transcardially perfused with 4% paraformaldehyde. Spinal cords were removed, postfixed overnight, and cut into 50-µm thickness coronal sections using a vibratome (Leica Microsysteme, Rueil-Malmaison, France). We used a standard Nissl staining protocol. Briefly, spinal cord sections were first dehydrated in standard series of increasing concentrations of alcohol (75%, 80%, 95%, 100%; 2 min each) and placed in distilled water for 5 mins. This step was followed by incubation of sections in a filtered solution containing 0.5% Cresyl violet (Sigma Aldrich) in distilled water, where they remained for approximately 1 min. Next, tissue was rehydrated by incubation in alcohol solutions of decreasing concentrations (100%, 95%, 70%, 50%; 2 min each) and cover-slipped.

## 3. Results

### 3.1. Partial Unilateral Dorsal Quadrant Lesion Entails Significant Neuronal Degeneration and Inflammation

**Figure 1:**
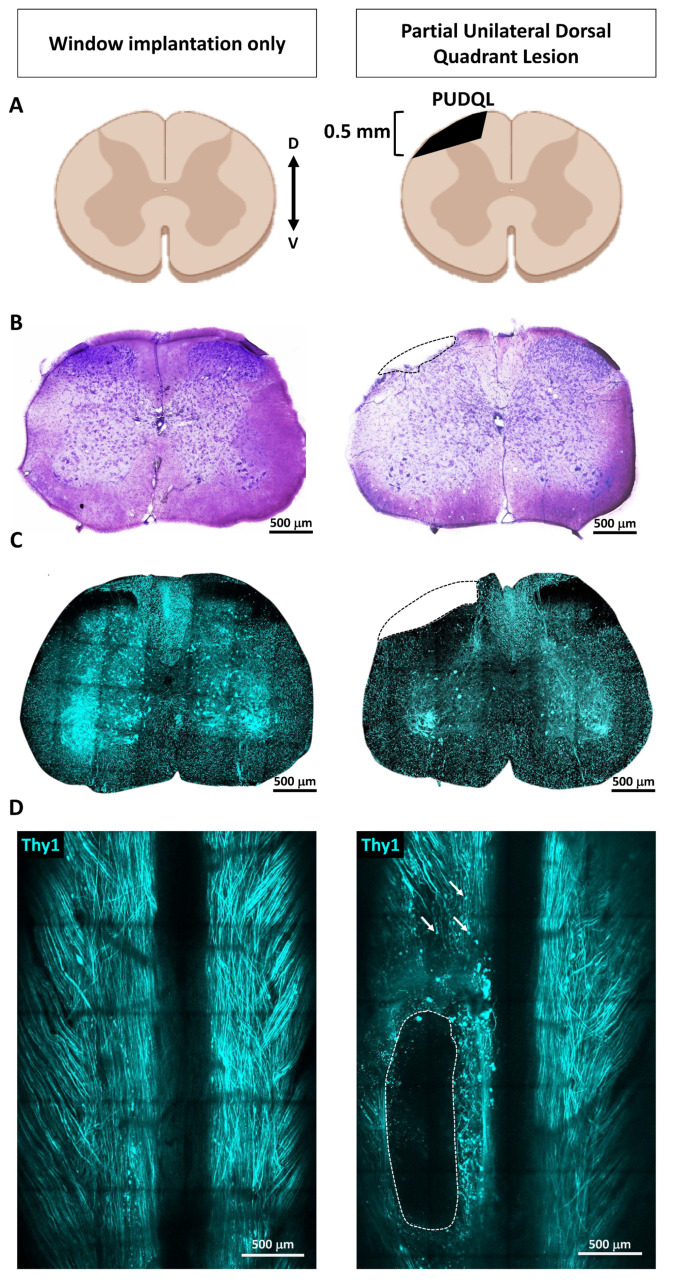
Spinal cord partial unilateral dorsal quadrant lesion (PUDQL) in Thy1-CFP mice. (**A**) Schematic view of the PUDQL (shadowed area in black). (**B**) Cresyl violet staining of transversal spinal cord section collected three days (D3) after window implantation or after PUDQL followed by window implantation. (**C**) Two-photon (2P) fluorescence images of spinal slices consecutive to the one of B). (**D**) Dorsal view of the same Thy1-CFP spinal cord imaged through the implanted window showing the primary lesion area (dotted lines) and examples of subsequent Wallerian degeneration of axons (arrows) on D3. D: dorsal; V: ventral.

**Figure 2:**
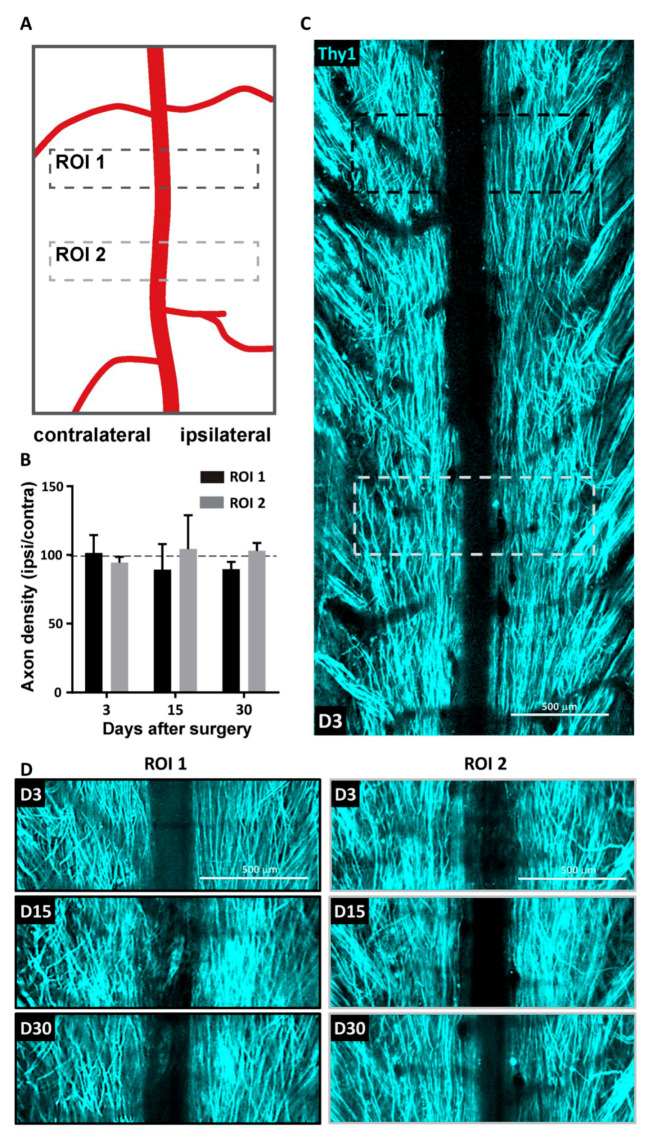
Absence of axonal degeneration following spinal cord window implantation. (**A**) Regions of interest (ROI) used to monitor the stability of axonal pattern over 30 days in unlesioned animals (**B**) Evolution of the axonal density over time. Density on one side was normalized to the contralateral side. Data are shown as mean ± SEM (n = 5). (**C**) Representative maximal intensity projection 2P images obtained *in vivo* three days (D3) after window implantation. (**D**) Zoom in images obtained 3, 14 and 30 days after window implantation for the same ROI1 (black) and ROI2 (gray) highlighted in (**C**).

We developed a partial unilateral dorsal quadrant lesion (PUDQL) as a model for SCI. Mice were equipped with a glass-window (see Materials and Methods) right after surgery to repeatedly image the same volume of interest (VOI) throughout the progression of the lesion. Observations were compared with those obtained on mice with window only and without lesion (Figure 1). The lesion is reproducible and suitable for comparing ipsi- and contralateral sides of the spinal cord. It is also minimally invasive and avoids unnecessary functional deficits, but remains large enough to appreciate a significant amount of neurodegeneration and inflammatory responses. Motor deficits were minimal, since the lesion severed only the most dorsal axon tracts (Figure 1B). Indeed, all mice were fully active the day after the injury and displayed exploratory behaviour. Our primary goal was to characterize the spatiotemporal axonal and inflammatory responses to the injury. To this end we took advantage of Thy1-CFP//LysM-EGFP//CD11c-EYFP triple transgenic mice (see Materials and Methods). In these mice, Thy1-CFP labels most projection neurons, including those located in the dorsal root ganglion (DRG), which project through the dorsal columns to supraspinal structures [22]. The PUDQL resulted in a severe reduction of CFP-labeled axons after the lesion (Figure 1C,D). Axon degeneration extended over 400 µm rostrally and up to 800 µm caudally from the center of the lesion (Figure 1D). No change in axon density was observed in unlesioned animals over time despite the window implantation surgery (Figure 2) [19].

**Figure 3:**
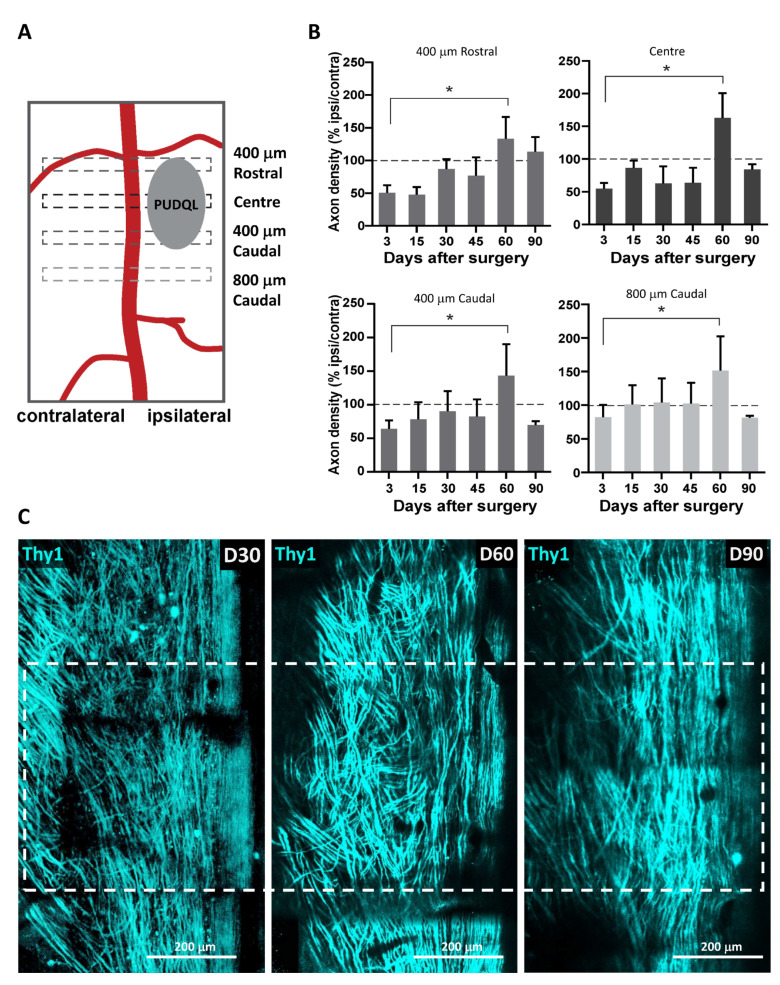
Spontaneous regeneration of axons following PUDQL. (**A**) Schematic representation of the regions of interest (ROIs) where axons were counted relatively to the location of the PUDQL. (**B**) Evolution of the normalized axonal density over 90 days after PUDQL (n = 5). (**C**) Representative intravital images of the same field of view centered on PUDQL. ROI corresponding to lesion epicenter is plotted (dotted square). Note the reduced axonal density at D30, the high density of tortuous axons at D60, as well as the dense network of straight axons by D90. The dense sprouting observed at the end of the second month was then pruned after three months. Data are shown as mean ± SEM. * *p* < 0.05.

In injured animals, axon density was measured at different distances from the centre of the lesion (Figure 3A). It decreased by approximately 50% when imaging data were collected at D0–3 at the centre of the lesion (Figure 3B). A similar reduction was observed 400 µm rostral but not 800 µm caudal to the lesion. Spontaneous axon sprouting occurred during the first two months after injury. At D60, axon densities increased beyond normal values and reached a 150% increase from before the injury (Figure 3B). The topography of these dorsal axons was also dramatically changed (Figure 3C). These axons that normally display a straight rostro-caudal alignment, strayed from their regular trajectories. A disorganized growth was particularly striking two months after the lesion (Figure 3C) and correlated with the high number of axon sprouts counted at that time (Figure 3B). Three months after lesion the number of axons recovered values similar to those observed before lesion and straighter trajectories were finally observed, particularly at the centre and rostral part of the lesion (Figure 3C, D90).

**Figure 4:**
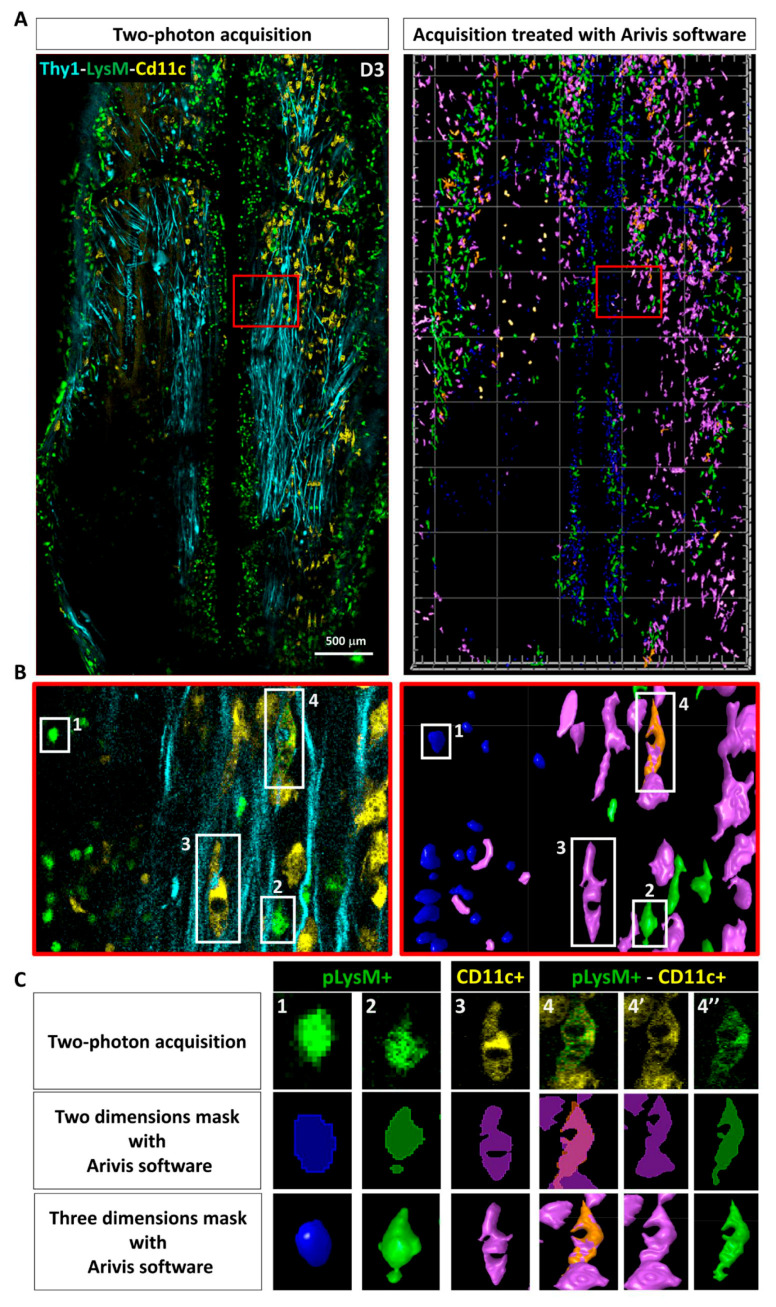
Automated image analysis pipeline used to characterize post-traumatic immune response. (**A**) Dorsal view obtained by intravital 2P microscopy 3 days after PUDQL in Thy1-CFP//LysM-EGFP//CD11c-EYFP triple transgenic mouse (left), and its corresponding 3D volume rendering following segmentation and labelisation of the cells using Arivis software (right). (**B**) Zoom in of the red squared area of (**A**) showing the four types of immune cells detected in the spinal axonal network. Raw data (left) and corresponding segmented images (right) (**C**) typical examples of each cell type presenting the raw 2P maximal intensity projection image, its 2D mask and 3D volume rendering obtained at the end of our Arivis analysis pipeline. 4-4’-4’’ show a double labelled pLysM+/CD11c+ cell in the multicolor image (4) as well as in each EYFP (4’) and EGFP (4’’) channels that were analysed independently prior to making mask intersection.

We used Arivis Vision 4D to set-up an automated image segmentation and analysis of immune cells in the response to PUDQL (Figure 4). Cells were automatically counted in two photon images stacks according to their fluorescence and morphologies. Four different cell subsets were identified from LysM-EGFP and CD11c-EYFP labelled cells (Figure 4A). Based on their respective morphological features, LysM-EGFP cells could be further categorized into circulating LysM+ cells (cLysM+; monocytes and neutrophils) and parenchymal LysM+ cells (pLysM+; monocytes derived cells). cLysM+ cells were round and motile, often found in perivascular regions, whereas pLysM+ were found in the spinal cord parenchyma and exhibited larger cytoplasmic extensions (Figure 4B,C). CD11c-EYFP (hereafter CD11c+) cells represented a population of activated microglia [20] (Figure 4B) that were located in the spinal cord parenchyma. These cells were morphologically similar to pLysM+ with ramified cytoplasmic extensions. Double-labeled cells (CD11c-EYFP+/LysM-EGFP+; Figure 4B) were previously identified by fluorescence-activated-cell-sorting as monocyte-derived dendritic cells (moDC) [20,23].

**Figure 5:**
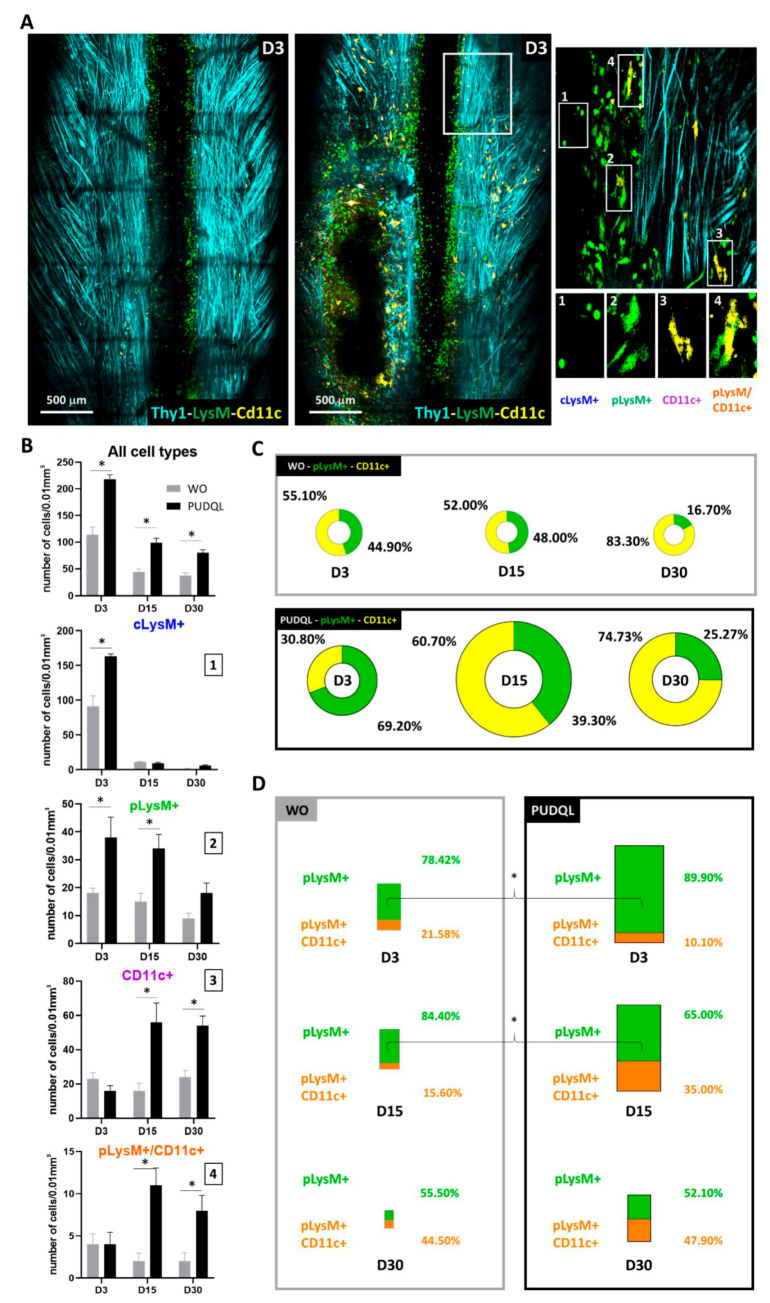
Characterisation of immune cells response after PUDQL in triple fluorescent mice. (**A**) Representative maximal intensity projection of 2P images obtained *in vivo* three days (D3) after PUDQL or window implantation only (WO). Note the massive axonal degeneration (dark area) underlined by immune cell accumulation following PUDQL (bottom left corner) in comparison to a contralateral area (top right white square). High magnification image of the white square showing the four identified cell types. (**B**) Quantitative evolution of global fluorescent cells densities (top) or individual subsets (1–4) in PUDQL (n = 5) and WO (n = 3) animals. Data are shown as mean ± SEM. (**C**) Circular graphs showing the relative percentage of pLysM+ and CD11c+ cells 3, 15 and 30 days after window implantation with or without PUDQL. Diameter of the circles is proportional to the total number of pLysM+ and CD11c+ cells densities quantified at each time point. (**D**) Bar graphs showing the relative percentage of pLysM+/CD11c+ cells among pLysM+ cells 3, 15 and 30 days after window implantation with or without PUDQL. Height of the bar is proportional to the total number pLysM+ and pLysM+/CD11c+. * *p* < 0.05.

When innate immune cells were taken together, irrespective of their subtypes, their numbers were about two-fold higher in PUDQL mice compared to mice without lesion at all measured time points (Figure 5B). The inflammatory response was initially carried by peripheral LysM+ cells in the first week (70%, Figure 5B,C) while CD11c+ cells and double-labelled cells showed a delayed increase from the second week onward (Figure 5B,C). On D3, the early increase of cLysM+ and pLysM+ cell densities were twice larger in PUDQL animals compared to unlesioned animals while the number of CD11c+ cells remained similar (Figure 5B). cLysM+ were only transient and quickly disappeared, while the density of pLysM+ cells remained high D15 and D30 after the lesion (Figure 5B). In PUDQL animals, D15 marked the start of the massive accumulations of CD11c+ cells and double-labelled cells to levels three to five-fold higher than in mice without lesion. The proportion of double-labelled cells increased with time to finally account for 48% of the pLysM+ density on D30 (Figure 5D).

Altogether, our results show that the implantation of a dorsal window triggers only a mild inflammatory response while the PUDQL model produces important neurodegeneration and inflammation that are suitable to evaluate the regenerative and immunomodulatory effects of MF implantation.

### 3.2. bf-MFs Are Biocompatible and Promote Axonal Sprouting

Biofunctionalized conducting polymer-coated carbon MFs have been proposed for fabricating regenerative neuroprosthetic devices [18,24]. We sought to determine whether MFs can be effectively implanted in the spinal cord after PUDQL injury using inflammation as a readout of their biocompatibility and axonal densities as a readout of their proregenerative efficacy.

**Figure 6:**
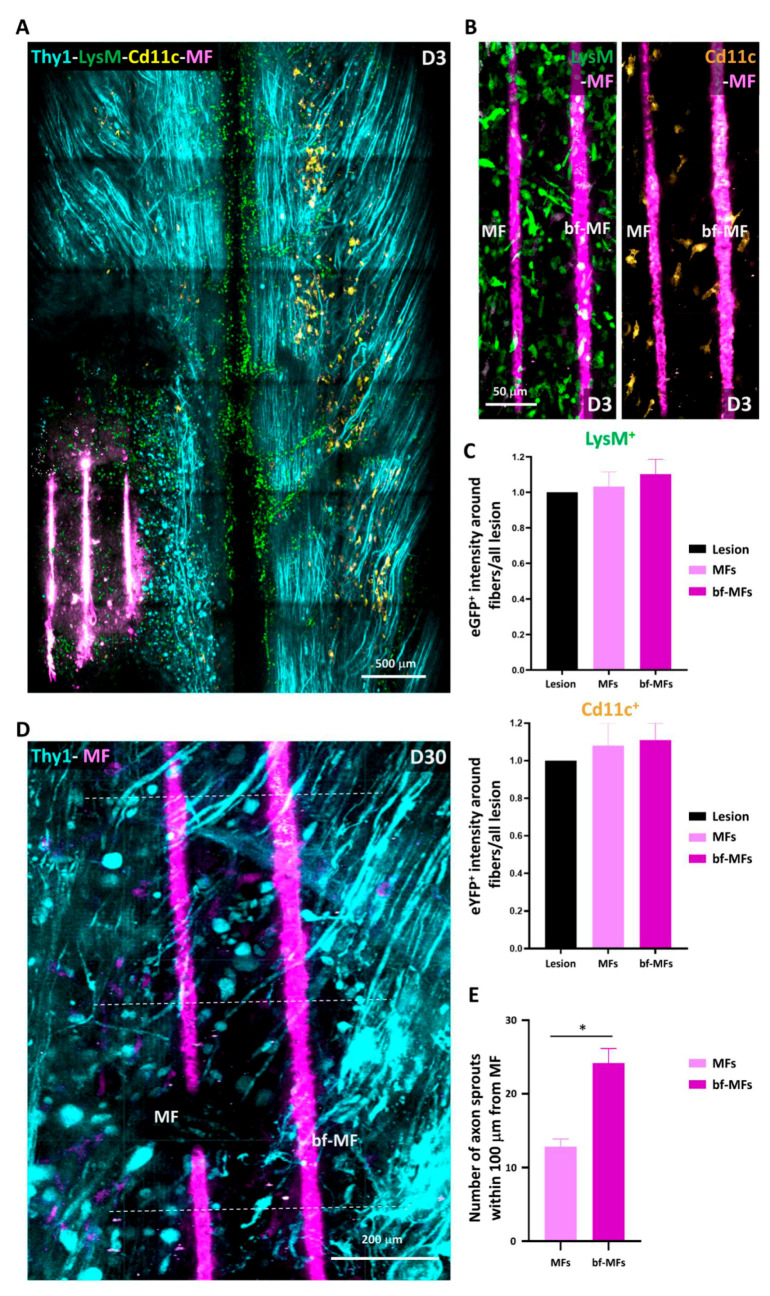
Biocompatibility of carbon microfibres and the effect of protein-coating. (**A**) Representative maximal intensity projection 2P images obtained *in vivo* three days after PUDQL followed by implantation of Microfibres (MFs, pink). Note the only mild accumulation of inflammatory cells inside the lesion. (**B**) Even distribution of LysM+ and CD11c+ in the vicinity of MFs three days after PUDQL. Note the absence of accumulation on the surface of MF in the presence (bf-MF) or absence (MF) of protein coating. (**C**) Average fluorescence intensity of EGFP or EYFP in a 100 µm box centered on the MFs. Fluorescence was normalized to the average level encountered in the whole lesioned area. (n_bf_ = 13, n_bare_ = 9 out of 7 mice) (**D**) High magnification image of the lesion site 30 days after PUDQL. Axon density was higher in the vicinity of coated MFs (bf-MF) than non-coated ones (MFs), in particular at levels where axons sprouts were counted (dotted lines). (**E**) Average number of axons sprouts counted within the first 100 µm from the bf-MF or MF surface. Data are shown as mean ± SEM (n_bf_ = 11; n_bare_ = 6).* *p* < 0.05.

To do so, bare-MFs and bf-MFs were manually inserted in alternation inside the spinal cord lesion following the rostro-caudal axis (Figure 6A). Irrespective of whether the fibres were protein-coated or not, they triggered minimal inflammatory reactions (Figure 6A,B). These reactions were in no case larger than in PUDQL-only animals (Figure 5A). No preferential accumulation of inflammatory cells was observed in the vicinity of either MF subtype in such traumatic environment (Figure 6B), as already reported in rats [17] Microglia and granulocytes/monocytes densities were evenly distributed in both MF subtypes (Figure 6C). These fibres are therefore biocompatible and suitable for implantation within the spinal cord.

The protein coating in bf-MFs has been reported to promote axonal elongation at least through the interaction with glial cells [17,18]. We evaluated the density of Thy1-CFP-axons in the vicinity of implanted bare and bf-MFs and observed a two-fold higher density of axon sprouts in a 100 µm vicinity of bf-MFs compared to bare-MFs (Figure 6D,E). This bias was observed throughout the first 30 days post-implantation, suggesting that axons were able to recognize and transduce the survival and growth signals borne by the coated molecules. bf-MFs were then preferred for subsequent experiments and referred to under the general term MF.

### 3.3. Effect of MF Implantation on Axon Growth and Guidance

**Figure 7:**
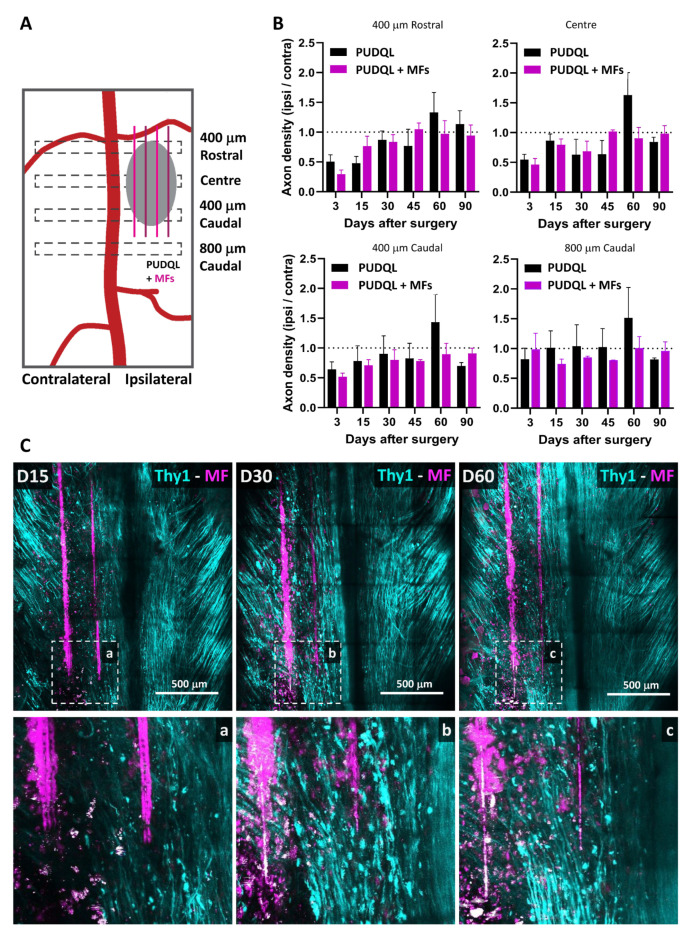
Effects of MFs on axon growth and guidance. (**A**) Schematic representation of the regions where axons were counted relatively to the location of the PUDQL+ MFs. (**B**) Comparative evolution of the normalized axonal density over 90 days after PUDQL (black; n = 5) or PUDQL followed by MF implantation (pink; n = 5). The increased sprouting observed on day 60 (D60) in PUDQL mice was greatly reduced with MF implantation. Data are shown as mean ± SEM. (**C**) Representative images of the same region of the spinal cord 15, 30 and 60 days following PUDQL. High magnification image of the same region outlined by a dotted square (bottom a, b and c). Note that the damaged axonal network at D30 was replaced by a dense and straight array of axons gradually regenerating and densifying along MFs over two months.

We evaluated the global effect of MF implantation on Thy1-CFP axonal networks at the lesion site over time in comparison to PUDQL-only mice (Figure 7A). Similar patterns in the evolution of axon density were observed in MF-bearing and PUDQL-only mice during the first month after injury (Figure 7B). After an initial decrease (~50%) in axon densities at D0–3, axon numbers at the lesion site increased gradually from D15–30 and reached normal values by D45. Interestingly, at D60, the axonal density in MF-implanted mice was 90% of control at the center of the lesion, whereas there was a 163% increase in mice with PUDQL-only. Although this effect was particularly significant at the centre of the lesion, it was also observed 400 µm away both rostrally and caudally. Axonal pruning took place (Figure 3C) and axonal density recovered normal values by D90 in PUDQL-only mice (Figure 7B).

These densities were achieved as early as D60 in MF implanted animals, where MFs in fact served as guiding scaffolds for axons. Indeed, Thy1-CFP axons were aligned along the fibres and their trajectories clearly differed from those observed in PUDQL-only mice. While axons deviated from their rostro-caudal trajectories and displayed random orientations in PUDQL animals (Figure 3C), axons showed directed growth, were straighter and longitudinally aligned in MF-implanted animals (Figure 7C). In these animals, we also did not observe an increase in axon densities beyond normal values, as if aberrant axonal sprouting was constrained by the guidance role exerted by the fibres. Altogether, these results suggest that MFs stimulate early axon growth but also provide scaffolding effects, which guide axons to their normal trajectories.

### 3.4. MF Implantation Accelerates Remission from Inflammation

**Figure 8:**
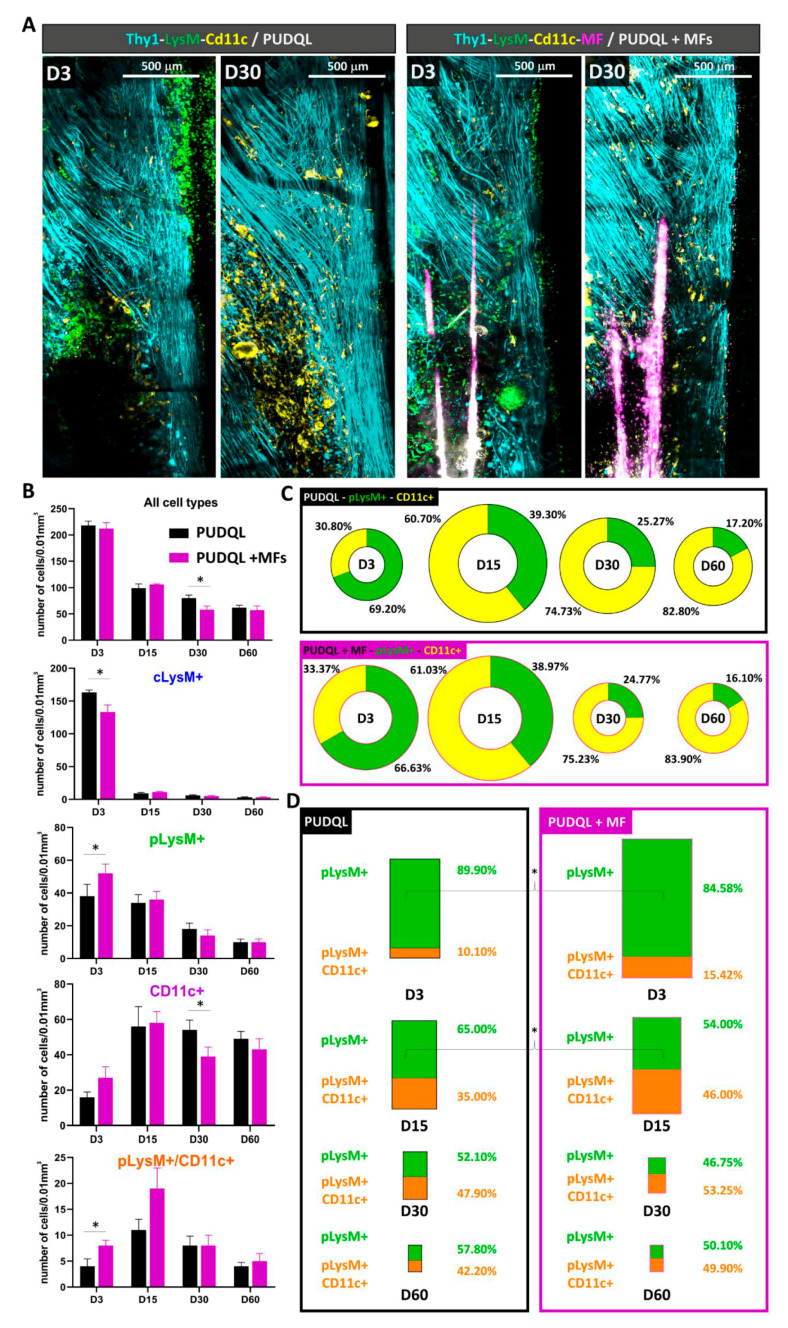
Effect of MFs implantation on post-traumatic inflammation. (**A**) Representative images of the same hemi-spinal cord three days (D3) and 30 days (D30) after PUDQL or PUDQL +MF implantation. (**B**) Quantitative evolution of fluorescent cells densities considered as a whole (top) or as individual color coded subsets in PUDQL (n = 5) and PUDQL + MFs (n = 5) animals. Data are shown as mean ± SEM. (**C**) Circular graphs showing the relative percentage of pLysM+ and CD11c+ cells 3, 15, 30 and 60 days after PUDQL or PUDQL+MF implantation. Diameter of the circles is proportional to the total number of pLysM+ and CD11c+ cells densities quantified at each time point. (**D**) Bar graphs showing the relative percentage of pLysM+/CD11c+ cells among pLysM+ cells 3, 15, and 30 and 60 days after PUDQL or PUDQL+MF implantation. Height of the bar is proportional to the total number pLysM+ and pLysM+/CD11c+. * *p* < 0.05.

Having initially verified that the MFs do not enhance tissue injury by deleterious inflammatory responses, we assessed whether MF implantation impacts the inflammatory reaction that develops after PUDQL. We quantified the densities of inflammatory cells throughout the progression of the injury (Figure 8). Globally, the total densities of fluorescent immune cells decreased over time in all injured mice (Figure 8B). These densities were not significantly different between MF-implanted mice and lesion-only mice at early time points (D0–3 and D15) (Figure 8B). At D30 however, the inflammatory response was significantly reduced in MF-implanted mice (57.7 ± 6.8 cells/vol; versus 80.0 ± 5.8 cells/vol; Kruskal-Wallis = 9.7, *p* = 0.0006). Total fluorescent cell density was decreased to similar numbers encountered a month later in absence of MFs (61.6 ± 4.6 PUDQL and 56.5 ± 8.1 PUDQL + MF at D60) and remained constant afterwards. These results show that the presence of MFs decreases the intensity of the inflammation and possibly accelerates the decrease of inflammation after injury.

### 3.5. MF Implantation Promotes Early Differentiation of moDCs

As a possible explanation for this acceleration, we observed that MF implantation impacted the relative contribution of different immune cell subsets at different stages of the post-traumatic period (Figure 8B). On D3, the transient increase of myeloid cLysM+ cells in blood vessels was significantly reduced in presence of MF and balanced by a significant increase of pLysM+ cells in the parenchyma. Although MFs did not significantly impact the average recruitment or activation of resident CD11c+microglial cells on D3, their late accumulation was significantly reduced on D30. However, the relative contribution of resident EYFP+ microglia versus EGFP+ peripheral immune cells remained virtually unchanged at all times points in the two conditions (Figure 8C).

The most important immunomodulatory effect of MF was observed on moDCs, namely the double-labelled (LysM+/CD11c+) cells. The density of these cells was increased at D0-3 and D15 postinjury in MF-implanted mice in comparison to PUDQL lesion-only mice (Figure 8B). This subpopulation represented a larger population of total pLysM+ cells in MF-implanted mice in comparison to PUDQL-only animals, irrespective of the time point considered (Figure 8D). Levels close to 50% were achieved at least two weeks earlier than in absence of microfibres.

Altogether, these results suggest that MF implantation triggers a faster and larger differentiation of pLysM+ cells into moDCs that seems favourable to accelerate the remission from inflammation.

## 4. Discussion

The treatment strategies for SCI are shifting toward combinatorial approaches. The characteristics provided by biomaterial scaffolds [18,25,26,27,28] have led many studies to incorporate scaffolds into their treatment paradigms ( [14] for review). Among them, bio-electronic microsystems hold strong promise for repairing neural damage and enhancing functional recovery. However, their development has been delayed in part because such devices should be biocompatible and their implantation requires procedures that inflict additional lesion to the neural tissue. Therefore, animal models and technical approaches are needed to precisely evaluate at the cellular level their effects over time. We designed a preclinical model of SCI in mice to evaluate in vivo the dynamic effects on neurons and inflammatory cells upon insertion of carbon MFs. The PUDQL lesion we used in this study was compatible with MF implantation in the lesion while avoiding major motor deficits in mice. The carbon MFs were coated with the conducting polymer PEDOT: PSS-co-MA and functionalized with the multimolecular complex PLL/heparin/bFGF/FN. Our results showed that these fibres are biocompatible as their insertion into the lesion did not trigger the acute inflammatory response beyond that observed in the lesion alone. Interestingly, they impinged upon the profile of immune cells that were recruited at later phases after the lesion, which may have resulted from the modified axon behaviour. We implanted MFs with an orientation parallel to the rostro-caudal axis and observed that they acted as scaffolds, resulting in a better axon alignment compared to controls. Furthermore, the protein-functionalized-MFs at least transiently supported and enhanced the endogenous potential of axons to sprout during the early (acute and subacute) phases. Therefore, functionalized carbon MFs provide an encouraging strategy for repair after SCI. Moreover, our experimental settings proved to be instrumental to longitudinally visualize the effect of biomaterial insertion at the cellular and circuit level in individual animals.

Regardless of the pathophysiological relevance of mice models [29], we used these animals due to the availability of the transgenic Thy1-CFP//LysM-EGFP//CD11c-EYFP triple heterozygous animals that allow to concomitantly image several fluorescent cell populations. Compared to most of the biocompatibility studies reported so far, our 2P intravital approach allowed us to observe live cells and the same area in a given animal over time. We found that MF types were well-integrated in the spinal cord tissue and did not exacerbate inflammation in their vicinity irrespective of the presence of protein coating. Quantification of the density of subsets of innate immune cells could not evidence any significant differences or accumulation along fibres. Therefore, PEDOT:PSS-co-MA is a relatively inert material and is not rejected by the mouse immune system. Furthermore, PLL/heparin/bFGF/fibronectin coating did not specifically attract innate myeloid cells or activated microglia. On the other hand, we observed a differential effect of the fibres on the density of fluorescent sensory axons at early phases (D3 to D30 after lesion). Since neuron survival and axon sprouting depend on molecular cues in the surrounding environment, it is likely that the coated proteins on the bf-MFs directly or indirectly modified the signals addressed to the receptors of the severed axons [30,31].

It is well known that mouse axons regenerate spontaneously within the first month after SCI [6,32]. Even if axons with enhanced regenerative capacity are able to grow across a lesion using astrocytes as support [33] they fail to grow across a large lesion. While we observed significant sprouting of axons after the lesion, their trajectories greatly differed from their normal rostro-caudal orientation. MFs may then serve as bridges for axon elongation either directly or in association with glial cells, as MFs functionalized with PLL/Heparin/bFGF/FN have been shown to promote glial migration along the fibres and thus provide environmental cues for aligned growth [18]. This effect may be particularly important at early time points when the association of regrowing axons with MFs must be ensured. Afterwards, we could not exclude the possibility that the proteins coating the MFs were released or degraded. Nevertheless, the physical support they provided to the tissue at later time points seemed enough to guide axons in the right direction during regeneration, as has been shown for other biomaterial scaffolds [26,27]. It should be noted that MFs can be coated with very different combinations of proteins and thus offer an extensive therapeutic flexibility. Besides proteins associated to cell adhesion and growth factors, the MF coating could also provide modulators of molecular pathways involved in intrinsic growth, thereby extending the range of manipulation of the local environment, especially at early time points [33,34,35,36].

Inflammation plays a predominant role in the aftermath of SCI and subsequent axonal regeneration. Having characterized the phenotype of fluorescent cells subpopulations in our Thy1-CFP//LysM-EGFP//CD11c-EYFP mice [20,23], we showed that the neuroinflammatory response is generally mediated by various myeloid cell types including infiltrating neutrophils, monocytes and their progeny, as well as by resident microglia [20,32]. Here our glass window preparation in triple transgenic mice allowed us to follow the dynamic evolution of immune cells, along with axonal plasticity, as well as the changes in these dynamics induced by MF implantation. We observed that the early phase of PUDQL (D0-3) was governed by a predominance of peripheral pLysM+ inflammatory cells over resident CD11c+ microglia. This dominance progressively declined over time until the lesion environment reached an inverted state dominated by CD11c+microglia in later stages of the trauma. Resident CD11c+ and peripheral pLysM+ immune cell densities thus appeared to balance each other out, as observed in other neuropathological conditions [20,23,32]. In all these cases moreover, the highest relative densities of pLysM+ immune cells coincided with conditions of maximal neural degeneration, while the highest relative densities of CD11c+ microglia coincided with conditions of regeneration and healing of the neural tissue.

Upon MF implantation at early stages (D0-3), the participation of pLysM+ to the inflammatory response was higher than the one observed in PUDQL-only mice. Whether this early mobilisation could account for the accelerated reduction of inflammatory cell densities on D30 remains to be clarified. Noteworthy, CD11c+ microglial contribution stabilized to a level three times larger than the one of peripheral immune cells at the time of axonal network recovery. Altogether these observations suggested that post-traumatic immune response switched from a deleterious proinflammatory acute state mediated by the early recruitment of pLysM+ cells to a prohealing anti-inflammatory chronic state involving resident CD11c+ microglia.

Part of these pLysM+ cells are neutrophils which are the first reported peripheral immune cells to infiltrate the spinal cord by 3–6 h. They peak around 1-day post injury and their presence is short lived ([37] for review). The other part are monocytes which at later times represent the vast majority of pLysM+ cells [20].

In the presence of MFs, the decrease of cLysM+ cells was coincident with a slight increase of pLysM+ cells in the spinal cord tissue. Mechanical constraints exerted by MFs and subsequent release of damage associated molecular patterns along implantation tracks most likely accelerated the recruitment of peripheral immune cells. In response to the factors present in the microenvironment, monocytes then differentiated locally towards moDC as indicated by their double (EGFP+/EYFP+) labelling [20]. MoDCs subsequently regulate the adaptive immune response and are key in secondary events such as the recruitment of T- and B-lymphocytes [38]. In particular, they can exhibit anti-inflammatory properties through the education of regulatory T cells. For example, their adoptive transfer was shown to dampen autoimmune deficits in a model of Experimental Autoimmune Encephalomyelitis (EAE) [39]. In our PUDQL model, these cells represented ~10% of the overall pLysM+ population in the first few days post-trauma, but their contribution increased progressively to reach ~40% on D60, a time of presumed anti-inflammatory condition. Importantly, MF implantation was responsible for increasing their contribution by half as early as D3, while a ~50% contribution was already observed from D15 onwards. In all PUDQL animals, their proportion increased coincidently with the contribution of CD11c+ microglia, supporting the idea of mutual interactions that might induce resident microglia to express growth promoting factors associated with a prohealing phenotype such as neurotrophin-3 and thrombospondin ([29] for review).

## 5. Conclusions

In conclusion, inserting MFs emerges as a potential therapeutic approach not only to accelerate the decrease of the inflammatory response but also to induce a change in the magnitude of the microglial and moDC influences on the repair process. Whether moDC densities can be tuned linearly by inserting more MFs in the lesion remains an open question. Inserting bundles with large number of fibres might also maximise both the probability and the kinetics of axonal regeneration. MFs act as a physical scaffold able to guide and to improve axon alignment and to limit aberrant misrouted connections. Optimization of the current MF protein coating might finally lead to further acceleration of the axonal regeneration. Since electrical conductivity of carbon allows for flowing current into the MFs [21,25], our future studies aim to evaluate if electrically stimulating neuronal activity further potentiates the already observed axonal regeneration.

## Data Availability

The data presented in this study are available on request from the corresponding author.

## References

[1] Profyris C., Cheema S.S., Zang D., Azari M.F., Boyle K., Petratos S. (2004). Degenerative and regenerative mecha-nisms governing spinal cord injury. Neurobiol. Dis..

[2] Beattie M.S., Hermann G.E., Rogers R.C., Bresnahan J.C., McKerracher L., Doucet G., Rossignol S. (2002). Chapter 4 Cell death in models of spinal cord injury. Progress in Brain Research.

[3] Yang T., Dai Y., Chen G., Cui S. (2020). Dissecting the Dual Role of the Glial Scar and Scar-Forming Astrocytes in Spinal Cord Injury. Front. Cell. Neurosci..

[4] Sofroniew M.V. (2018). Dissecting spinal cord regeneration. Nat. Cell Biol..

[5] Brus-Ramer M., Carmel J.B., Chakrabarty S., Martin J.H. (2007). Electrical Stimulation of Spared Corticospinal Axons Augments Connections with Ipsilateral Spinal Motor Circuits after Injury. J. Neurosci..

[6] Dray C., Rougon G., Debarbieux F. (2009). Quantitative analysis by in vivo imaging of the dynamics of vascular and axonal networks in injured mouse spinal cord. Proc. Natl. Acad. Sci. USA.

[7] Jiang Y.-Q., Zaaimi B., Martin J.H. (2016). Competition with Primary Sensory Afferents Drives Remodeling of Corticospinal Axons in Mature Spinal Motor Circuits. J. Neurosci..

[8] Tan A.M., Chakrabarty S., Kimura H., Martin J.H. (2012). Selective Corticospinal Tract Injury in the Rat Induces Primary Afferent Fiber Sprouting in the Spinal Cord and Hyperreflexia. J. Neurosci..

[9] Zheng B., Lorenzana A.O., Ma L. (2019). Understanding the axonal response to injury by in vivo imaging in the mouse spinal cord: A tale of two branches. Exp. Neurol..

[10] Lorenzana A.O., Lee J.K., Mui M., Chang A., Zheng B. (2015). A Surviving Intact Branch Stabilizes Remaining Axon Architecture after Injury as Revealed by In Vivo Imaging in the Mouse Spinal Cord. Neuron.

[11] Elzinga K., Tyreman N., Ladak A., Savaryn B., Olson J., Gordon T. (2015). Brief electrical stimulation improves nerve regeneration after delayed repair in Sprague Dawley rats. Exp. Neurol..

[12] Al-Majed A.A., Neumann C.M., Brushart T.M., Gordon T. (2000). Brief Electrical Stimulation Promotes the Speed and Accuracy of Motor Axonal Regeneration. J. Neurosci..

[13] Koffler J., Zhu W., Qu X., Platoshyn O., Dulin J.N., Brock J., Graham L., Lu P., Sakamoto J., Marsala M. (2019). Biomimetic 3D-printed scaffolds for spinal cord injury repair. Nat. Med..

[14] Führmann T., Anandakumaran P.N., Shoichet M.S. (2017). Combinatorial Therapies after Spinal Cord Injury: How Can Biomaterials Help?. Adv. Healthc. Mater..

[15] Wang X., Liu X., Yu S., Wang X., Zhang S., Wu Q., Sun X., Mao H.-Q. (2016). Co-effects of matrix low elasticity and aligned topography on stem cell neurogenic differentiation and rapid neurite outgrowth. Nanoscale.

[16] Yuk H., Lu B., Lin S., Qu K., Xu J., Luo J., Zhao X. (2020). 3D printing of conducting polymers. Nat. Commun..

[17] Collazos-Castro J.E., García-Rama C., Alves-Sampaio A. (2016). Glial progenitor cell migration promotes CNS axon growth on functionalized electroconducting microfibers. Acta Biomater..

[18] Alves-Sampaio A., García-Rama C., Collazos-Castro J.E. (2016). Biofunctionalized PEDOT-coated microfibers for the treatment of spinal cord injury. Biomaterials.

[19] Fenrich K.K., Weber P., Hocine M., Zalc M., Rougon G., Debarbieux F. (2012). Long-term in vivo imaging of normal and pathological mouse spinal cord with subcellular resolution using implanted glass windows. J. Physiol..

[20] Caravagna C., Jaouen A., Desplat-Jégo S., Fenrich K.K., Bergot E., Luche H., Grenot P., Rougon G., Malissen M., Debarbieux F. (2018). Diversity of innate immune cell subsets across spatial and temporal scales in an EAE mouse model. Sci. Rep..

[21] Vara H., Collazos-Castro J.E. (2015). Biofunctionalized Conducting Polymer/Carbon Microfiber Electrodes for Ultrasen-sitive Neural Recordings. ACS Appl. Mater. Interfaces.

[22] Feng G., Mellor R.H., Bernstein M., Keller-Peck C., Nguyen Q.T., Wallace M., Nerbonne J.M., Lichtman J.W., Sanes J.R. (2000). Imaging Neuronal Subsets in Transgenic Mice Expressing Multiple Spectral Variants of GFP. Neuron.

[23] Ricard C., Tchoghandjian A., Luche H., Grenot P., Figarella-Branger M., Rougon G., Malissen M., Debarbieux F. (2016). Phenotypic dynamics of microglial and monocyte-derived cells in glioblastoma-bearing mice. Sci. Rep..

[24] Collazos-Castro J.E., Hernández-Labrado G.R., Polo J.L., García-Rama C. (2013). N-Cadherin- and L1-functionalised conducting polymers for synergistic stimulation and guidance of neural cell growth. Biomaterials.

[25] Vara H., Collazos-Castro J.E. (2019). Enhanced spinal cord microstimulation using conducting polymer-coated carbon microfibers. Acta Biomater..

[26] Li X., Zhang C., Haggerty A.E., Yan J., Lan M., Seu M., Yang M., Marlow M.M., Maldonado-Lasunción I., Cho B. (2020). The effect of a nanofiber-hydrogel composite on neural tissue repair and regeneration in the contused spinal cord. Biomaterials.

[27] Chedly J., Soares S., Montembault A., von Boxberg Y., Veron-Ravaille M., Mouffle C., Benassy M.N., Taxi J., David L., Nothias F. (2017). Physical chitosan microhydrogels as scaffolds for spinal cord injury restoration and axon regeneration. Biomaterials.

[28] Oudega M., Hao P., Shang J., Haggerty A.E., Wang Z., Sun J., Liebl D.J., Shi Y., Cheng L., Duan H. (2019). Validation study of neurotrophin-3-releasing chitosan facilitation of neural tissuegeneration in the severely injured adult rat spinal cord. Exp. Neurol..

[29] Alizadeh A., Dyck S.M., Karimi-Abdolrezaee S. (2019). Traumatic Spinal Cord Injury: An Overview of Pathophysiolo-gy, Models and Acute Injury Mechanisms. Front. Neurol..

[30] Cardozo M.J., Mysiak K.S., Becker T., Becker C.G. (2017). Reduce, reuse, recycle—Developmental signals in spinal cord regeneration. Dev. Biol..

[31] Webber C., Zochodne D. (2010). The nerve regenerative microenvironment: Early behavior and partnership of axons and Schwann cells. Exp. Neurol..

[32] Fenrich K.K., Weber P., Rougon G., Debarbieux F. (2013). Long- and short-term intravital imaging reveals differential spatiotemporal recruitment and function of myelomonocytic cells after spinal cord injury. J. Physiol..

[33] Zukor K., Belin S., Wang C., Keelan N., Wang X., He Z. (2013). Short hairpin RNA against PTEN enhances regenera-tive growth of corticospinal tract axons after spinal cord injury. J. Neurosci..

[34] Sun F., Park K.K., Belin S., Wang D., Lu T., Chen G., Zhang K., Yeung C., Feng G., Yankner B.A. (2011). Sustained axon regeneration induced by co-deletion of PTEN and SOCS3. Nat. Cell Biol..

[35] Kim C., Kim H.J., Lee H., Lee H., Lee S.J., Lee S.T., Yang S.R., Chung C.K. (2019). Mesenchymal Stem Cell Trans-plantation Promotes Functional Recovery through MMP2/STAT3 Related Astrogliosis after Spinal Cord Injury. Int. J. Stem Cells.

[36] Wu W.D., Wang L.H., Wei N.X., Kong D.H., Shao G., Zhang S.R., Du Y.S. (2019). MicroRNA-15a inhibits inflam-matory response and apoptosis after spinal cord injury via targeting STAT3. Eur. Rev. Med. Pharmacol. Sci..

[37] Summers C., Rankin S.M., Condliffe A.M., Singh N., Peters A.M., Chilvers E.R. (2010). Neutrophil kinetics in health and disease. Trends Immunol..

[38] Jones T.B. (2014). Lymphocytes and autoimmunity after spinal cord injury. Exp. Neurol..

[39] Kaushansky N., Kaminitz A., Allouche-Arnon H., Ben-Nun A. (2019). Modulation of MS-like disease by a multi epitope protein is mediated by induction of CD11c+CD11b+Gr1+ myeloid-derived dendritic cells. J. Neuroimmunol..

